# Life satisfaction 18 months and 10 years following spinal cord injury: results from a New Zealand prospective cohort study

**DOI:** 10.1007/s11136-022-03313-w

**Published:** 2023-01-26

**Authors:** Ruby Dixon, Sarah Derrett, Ari Samaranayaka, Helen Harcombe, Emma H. Wyeth, Carolyn Beaver, Martin Sullivan

**Affiliations:** 1 Department of Preventive and Social Medicine, Dunedin School of Medicine, University of Otago, Dunedin, New Zealand; 2 Division of Health Sciences, Ngāi Tahu Māori Health Research Unit, University of Otago, Dunedin, New Zealand; 3 Division of Health Sciences, Biostatistics Centre, University of Otago, Dunedin, New Zealand; 4 School of Social Work, Massey University, Palmerston North, New Zealand

**Keywords:** Life satisfaction, Spinal cord injury, Disability, Health-related quality of life

## Abstract

**Purpose:**

To examine the life satisfaction outcomes after spinal cord injury (SCI) and to identify the factors associated with life satisfaction at 18 months and 10 years post-SCI in New Zealand (NZ).

**Methods:**

Adults (16–64 years) were recruited between 2007 and 2009 from NZ’s two spinal units following first admission for SCI. Interviews at 6 months, 18 months, and 10 years post-SCI examined demographic, physical, psychosocial, economic, and environmental characteristics. Multivariable regression models were used to identify predictors of life satisfaction at each timepoint.

**Results:**

Overall, 118 people participated at 6 months, 103 at 18 months, and 63 at 10 years post-SCI. Pre-SCI, 90% of participants were satisfied with life, 67% were satisfied at 18 months, and 78% at 10 years. At 18 months post-SCI, participants who reported: never or sometimes using a wheelchair, no problems with self-care, no problems with anxiety or depression, no/lesser disability, or fewer secondary health conditions (SHCs) at 6 months post-SCI were more likely to be satisfied (*p* < 0.05), compared to those without these characteristics. Participants who experienced considerable disability at 6 months post-SCI were 22% less likely to be satisfied 10 years post-SCI compared to those experiencing no/lesser disability (*p* = 0.028).

**Conclusions:**

A higher proportion of participants were satisfied at both 18 months and 10 years post-SCI than not satisfied. To improve the likelihood of satisfaction with life, increased focus on reducing disability and providing supports for those using wheelchairs, experiencing anxiety/depression or problems with self-care, and effects of SHCs are promising for future potential interventions.

## Introduction

Spinal cord injuries (SCI) result from neurological damage to the spinal cord causing temporary or permanent change in function [1]. This can result in partial or complete loss of function below the site of the SCI [1, 2]. SCI can result from traumatic (injury) or non-traumatic causes (e.g. haemorrhage or cancer) [2].

The World Health Organization estimates the annual global incidence of SCI to be 40–80 million[3]. In New Zealand (NZ), the incidence is approximately 160 people per year [4]. In NZ, rates differ by ethnicity, with higher rates among Māori and Pacific people (46 and 70 per million, respectively) compared to European and other ethnicities (29 and 16 per million, respectively) [5, 6].

NZ has a unique no-fault injury insurance scheme, the Accident Compensation Corporation (ACC), which provides rehabilitation and financial support (up to 80% of their income) to people experiencing traumatic SCI (and other injuries) [7–9]. Non-traumatic SCI are covered by the general public health system with access to limited means-tested financial support [7, 8]. These different systems can lead to differences in treatment and experiences between those with traumatic and non-traumatic SCI [8].

SCIs can have large impacts on people’s lives, including on levels of life satisfaction [7, 10, 11]. Life satisfaction has been described as the degree to which people positively perceive their overall quality of life, state of mind, and contentment with the life they lead [12].

Previous research internationally found life satisfaction to be generally positive among people with SCI, although not as satisfied as the general population [13–16]. A longitudinal study from the United States followed 2183 people from one to 20 years post-SCI and found life satisfaction increased as time post-SCI increased [17]. Post-SCI life satisfaction has been associated with a range of factors including socio-demographic and SCI-related characteristics, physical and mental health, and social and environmental characteristics [18–21]. However, none of these relationships appear to have been explored in NZ where the health and ACC system is unique. It is important to identify the opportunities for interventions to improve life satisfaction for people after SCI in NZ. Therefore, the overall aim of this paper is to examine the post-SCI life satisfaction outcomes over time and to identify the factors associated with life satisfaction at 18 months and 10 years post-SCI in NZ.

## Methods

### Study design and participants

This research analyses the data previously collected by the ‘Longitudinal study of the life histories of people with spinal cord injury’ [7]. The study was led by a researcher with lived experience of SCI (MS), interviewers had lived experience (including CB), and questionnaires were developed in consultation with others with lived experience of SCI. Details of this study have been previously published [7, 22], but are briefly outlined below.

Individuals were recruited between 2007 and 2009 from NZ’s two spinal units following first admission for SCI. Participants were NZ citizens or permanent residents aged 16–64 years who had sustained traumatic or non-traumatic SCI. Eligible participants had an American Spinal Injury Association Impairment Scale (AIS) grade of A, B, C, or D [23]. AIS A is the most severe (and complete) grade SCI, followed by AIS B, C, and D [23]. People with AIS grade E, those with significant cognitive injury or communication impairment, or a life expectancy of less than six months at the time of their SCI were ineligible. All participants provided informed consent at the beginning of the study, and before each follow-up interview, and received a copy of the consent form for their records. Ethical approval was granted by the New Zealand Multi-region Ethics Committee (MEC07/09/117).

### Data collection

Data were collected from structured interviews undertaken approximately 6 months, 18 months, and 10 years post-SCI. Clinical information was collected by research nurses in each spinal unit. Interviews collected information about a range of demographic, physical, psychosocial, economic, and environmental characteristics. The first interview asked about pre-SCI characteristics as well as characteristics at 6 months post-SCI; subsequent interviews explored outcomes at later timepoints (the outcome of interest is life satisfaction).

**Fig. 1:**
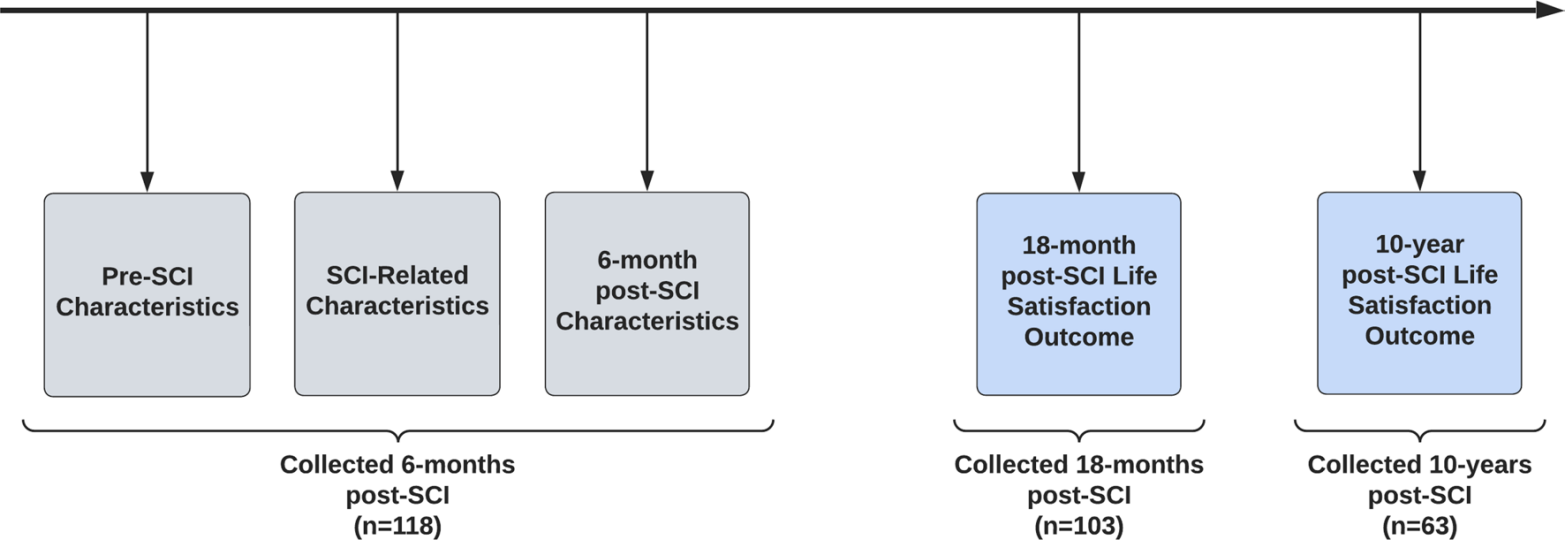
Data collection timepoints and interview participation

Overall, 118 people participated in a baseline interview (6 months post-SCI), 103 at 18 months post-SCI, and 63 at 10 years post-SCI (Fig. 1).

### Outcome measure

Life satisfaction outcomes at 18 months and 10 years were assessed by asking participants “Overall, how would you rate your life as a whole” on a 5-item scale [24, 25]. Responses were categorised as ‘Satisfied’ (“Completely satisfied” or “Mostly satisfied”) or ‘Not satisfied’ (“Neither satisfied nor dissatisfied”, “Mostly dissatisfied” or “Completely dissatisfied”).

### Potential predictors of life satisfaction

Potential predictors of post-SCI life satisfaction were identified from previous literature and categorised as pre-SCI, SCI-related, and 6-month post-SCI characteristics.

Pre-SCI characteristics included the following: age [26], sex [27], ethnicity (prioritised as per standard guidelines as “Māori”, “Pacific Peoples”, “Asian”, or “European/Other”) [27, 28], relationship status [26], living arrangements [27], highest educational qualification [26, 27], paid employment [26, 27], job security, adequacy of household income [5, 29], prior chronic conditions [7, 30], prior injuries [31, 32], prior long-term disability [27], family/whānau involvement, life satisfaction [24, 25], health-related quality of life (HRQoL) [33], and cognitive function [34].

SCI-related characteristics included the following: AIS grade (A, B, C, or D) [23], SCI aetiology (injury or illness), wheelchair usage (always, sometimes, or never), and ACC support (yes or no).

The 6-month post-SCI characteristics included the following: job security (very secure/secure or insecure/very insecure), HRQoL [33], cognitive function (no/any problems) [34], disability [35], secondary health conditions (SHCs) [26], satisfaction with social support [36], and sexual activity (decreased or not).

Life satisfaction was not assessed (as a potential predictor) at 6 months because there was concern (from people with lived experience) that this was too sensitive a question early post-SCI.

#### Measures

HRQoL was measured using the EQ-5D-3L dimensions of mobility, self-care, usual activities, pain or discomfort, and anxiety or depression [33]. Each dimension has three response options (e.g. no, some or extreme problems), categorised as ‘No’ or ‘Any’ problems. Participants also rated their own health state on the EQ-5D-3L Visual Analog Scale (VAS), where 0 is the worst health and 100 is the best health [33]. VAS scores were reported as a continuous variable.

Disability was measured by the 12-item World Health Organization Disability Assessment Schedule (WHODAS 2.0 12-item) [35]. Each item was allocated a score between 0 (none) and 4 (extreme/cannot do) to provide a total score between 0 (no disability) and 48 (maximum disability) [37]. Scores were dichotomised (‘No/lesser disability’ WHODAS score < 10; ‘Considerable disability’ WHODAS ≥ 10) [37].

Participants self-reported whether they had “any problems” with SHCs from a list: leg swelling, leg spasms, shortness of breath, difficulty coughing, bowel constipation, diarrhoea, indigestion, urinary tract infection, urinary incontinence, problems with bladder programme, headaches, back pain, shoulder pain, pain below the level of SCI, and ‘other’ problems [26, 38]. The number of SHCs with “any problems” for each participant was summed.

Satisfaction with social support was measured using five questions from the Social Support Survey Instrument [36]. Questions asked: “Is there someone available to you whom you can count on to listen when you need to talk?”, “Is there someone available to you to give you good advice about a problem?”, “Is there someone available to you who shows you love and affection?”, “Can you count on anyone to provide you with emotional support (talking over problems or helping you make a difficult decision)?”, and “Do you have as much contact as you would like with someone you feel close to, someone in whom you can trust and confide?” [36]. Responses ranged from “None of the time” (1) to “All of the time” (5). A summed score ranged between 5 and 25 and was treated as a continuous variable.

### Data analysis

Descriptive statistics [*n*, % for categorical variables; mean ± standard deviation (SD) for continuous variables] were calculated for pre-SCI, SCI-related, and 6-month post-SCI characteristics. Univariable analyses assessed associations between potential predictors and life satisfaction outcomes at 18 months and 10 years post-SCI. Chi-squared tests (or Fisher's exact tests where appropriate) were used to assess the association between categorical variables and life satisfaction; unpaired t-tests were used for continuous variables.

A multivariable modified Poisson regression model [39] was developed to identify the potential predictor variables associated with life satisfaction outcomes at 18 months and 10 years post-SCI separately. The variables used for these models were those with evidence of univariable association based on *p *values < 0.15, and variables deemed important based on prior knowledge and research. The aim was to produce separate ‘final multivariable models’ at 18 months and 10 years post-SCI. Due to the relatively small sample size, which can cause model over-fitting resulting in potentially missed influential variables from the two ‘final models’, we first created four ‘mini-models’: (1) demographic and pre-SCI characteristics, (2) SCI-related characteristics, (3) 6-month post-SCI EQ-5D-3L characteristics, and (4) other 6-month post-SCI characteristics. Backwards stepwise selection, with a *p* value < 0.15, was used for variable retention in the mini-models. Variables retained in these mini-models were used for building the final model. The backwards stepwise selection process was repeated in the final model; a *p *value < 0.30 was used considering the sample size and the number of parameters to be estimated. Stata version 16.1 was used for all analyses [40].

## Results

Table 1 describes the study participants at each of the three timepoints according to their pre-SCI, SCI-related, and 6-month post-SCI characteristics.

**Table 1:** Pre-SCI, SCI-related, and 6-month post-SCI characteristics of participants interviewed at each data collection point

Characteristics	6-month participants (*n* = 118)	18-month participants (*n* = 102)	10-year participants (*n* = 63)
	*n* (%)	*n* (%)	*n* (%)
**Pre-SCI Characteristics**			
Age at onset of SCI (years)			
Mean ± SD	41.0 ± 14.3	41.5± 14.5	42.2 ± 14.2
16–24	21 (17.8)	19 (18.6)	10 (15.9)
25–34	21 (17.8)	17 (16.7)	13 (20.6)
35–44	24 (20.3)	20 (19.6)	9 (14.3)
45–54	28 (23.8)	23 (22.5)	17 (27.0)
55–64	24 (20.3)	23 (22.5)	14 (22.2)
Sex			
Male	90 (76.3)	78 (76.5)	47 (74.6)
Female	28 (23.7)	24 (23.5)	16 (25.4)
Ethnicity			
Māori	23 (19.5)	16 (15.7)	7 (11.0)
Pacific Peoples	10 (8.5)	7 (6.9)	2 (3.2)
Asian	6 (5.1)	5 (4.9)	3 (4.8)
European	79 (66.9)	74 (72.5)	51 (81.0)
Relationship status**			
Married/Living with partner	64 (54.2)	60 (58.8)	39 (61.9)
Single	35 (29.7)	29 (28.4)	15 (23.8)
Separated/divorced/widowed	17 (14.4)	13 (12.8)	9 (14.3)
Living arrangements**			
With family	86 (72.9)	78 (76.5)	51 (81.0)
With non-family	18 (15.2)	15 (14.7)	7 (11.1)
Alone	10 (8.5)	8 (7.8)	5 (7.9)
Highest educational qualification**			
No qualification	34 (28.8)	27 (26.5)	13 (20.6)
School	21 (17.8)	20 (19.6)	14 (22.2)
Post-secondary	59 (50.0)	53 (52.0)	35 (55.6)
Paid employment			
≥ 30 hours	92 (78.0)	79 (77.5)	51 (81.0)
< 30 hours	6 (5.1)	5 (4.9)	2 (3.2)
Not in paid employment	20 (16.9)	18 (17.6)	10 (15.8)
Job security**			
Very secure/secure	87 (73.7)	75 (73.5)	48 (76.2)
Insecure/very insecure	7 (5.9)	6 (5.9)	4 (6.4)
Adequacy of household income**			
More than enough/enough	82 (69.5)	75 (73.5)	50 (79.4)
Just enough/not enough	32 (27.1)	25 (24.5)	13 (20.6)
Prior chronic conditions			
No	72 (61.0)	59 (57.8)	35 (55.6)
Yes	46 (39.0)	43 (42.2)	28 (44.4)
Prior injuries**			
No	96 (81.4)	82 (80.4)	50 (79.4)
Yes	21 (17.8)	19 (18.6)	12 (19.1)
Prior long-term disability**			
No	102 (86.4)	86 (84.3)	56 (88.9)
Yes	15 (12.7)	15 (14.7)	7 (11.1)
Family/whānau involvement**			
Very large part/large part	104 (88.1)	92 (90.2)	58 (92.1)
Small part/very small part	12 (10.2)	10 (9.8)	5 (7.9)
Pre-SCI life satisfaction			
Satisfied	106 (89.8)	93 (91.2)	58 (92.1)
Not satisfied	12 (10.2)	9 (8.8)	5 (7.9)
EQ-5D-3L Mobility			
No problems	106 (89.8)	91 (89.2)	58 (92.1)
Any problems	12 (10.2)	11 (10.8)	5 (7.9)
EQ-5D-3L Self-Care			
No problems	115 (97.5)	99 (97.1)	62 (98.4)
Any problems	3 (2.5)	3 (2.9)	1 (1.6)
EQ-5D-3L Usual Activities			
No problems	110 (93.2)	95 (93.1)	59 (93.7)
Any problems	8 (6.8)	7 (6.9)	4 (6.3)
EQ-5D-3L Pain or Discomfort			
No problems	96 (81.4)	81 (79.4)	51 (81.0)
Any problems	22 (18.6)	21 (20.6)	12 (19.0)
EQ-5D-3L Anxiety or Depression			
No problems	104 (88.1)	91 (89.2)	57 (90.5)
Any problems	14 (11.9)	11 (10.8)	6 (9.5)
EQ-5D-3L 0–100 VAS			
Mean ± SD	86.0 ± 18.2	85.4 ± 18.0	85.4 ± 15.9
Cognitive function			
No problems	107 (90.7)	94 (92.2)	58 (92.1)
Any problems	11 (9.3)	8 (7.8)	5 (7.9)
**SCI-related Characteristics**			
AIS grade			
A	36 (30.5)	32 (31.4)	16 (25.4)
B	9 (7.6)	6 (5.9)	2 (3.2)
C	10 (8.5)	10 (9.8)	8 (12.7)
D	63 (53.4)	54 (52.9)	37 (58.7)
SCI aetiology			
Injury	91 (77.1)	79 (77.5)	48 (76.2)
Illness	24 (20.3)	20 (19.6)	14 (22.2)
Don’t know	3 (2.6)	3 (2.9)	1 (1.6)
Wheelchair usage			
Yes	59 (50.0)	51 (50.0)	26 (41.3)
No	45 (38.1)	39 (38.2)	30 (47.6)
Sometimes	14 (11.9)	12 (11.8)	7 (11.1)
ACC support			
Yes	93 (78.8)	81 (79.4)	50 (79.4)
No	25 (21.2)	21 (20.6)	13 (20.6)
**6-month post-SCI Characteristics**			
Job security**			
Very secure/secure	38 (32.2)	36 (35.3)	28 (44.4)
Insecure/very insecure	21 (17.8)	15 (14.7)	10 (15.9)
Not applicable	34 (28.8)	29 (28.4)	13 (20.6)
EQ-5D-3L Mobility			
No problems	9 (7.6)	8 (7.8)	8 (12.7)
Any problems	109 (92.4)	94 (92.2)	55 (87.3)
EQ-5D-3L Self-Care			
No problems	40 (33.9)	37 (36.3)	26 (41.3)
Any problems	78 (66.1)	65 (63.7)	37 (58.7)
EQ-5D-3L Usual Activities			
No problems	12 (10.2)	11 (10.8)	6 (9.5)
Any problems	106 (89.8)	91 (89.2)	57 (90.5)
EQ-5D-3L Pain or Discomfort			
No problems	16 (13.6)	13 (12.7)	8 (12.7)
Any problems	102 (86.4)	89 (87.3)	55 (87.3)
EQ-5D-3L Anxiety or Depression**			
No problems	59 (50.0)	54 (52.9)	34 (54.0)
Any problems	58 (49.2)	47 (46.1)	28 (44.4)
EQ-5D-3L 0–100 VAS			
Mean ± SD	54.1 ± 22.2	55.6 ± 21.2	56.1 ± 20.8
Cognitive function			
No problems	67 (56.8)	62 (60.8)	38 (60.3)
Any problems	51 (43.2)	40 (39.2)	25 (39.7)
Disability (WHODAS)			
No/lesser disability (0–9)	24 (20.3)	22 (21.6)	15 (23.8)
Considerable disability (≥10)	94 (79.7)	80 (78.4)	48 (76.2)
SCI-related SHCs			
Mean ± SD	5.8 ± 2.9	5.8 ± 2.8	6.1 ± 2.6
Satisfaction with social support			
Mean ± SD	21.6 ± 3.5	21.7 ± 3.6	21.5 ± 3.8
Sexual activity decreased**			
No	24 (20.4)	22 (21.6)	13 (20.6)
Yes	88 (74.6)	76 (74.5)	48 (76.2)
Not applicable	3 (2.5)	3 (2.9)	2 (3.2)

*ACC* Accident Compensation Corporation; *AIS* American Spinal Injury Association Impairment Scale; *SCI* spinal cord injury; *SHCs* secondary health conditions; *VAS* Visual Analog Scale; *WHODAS* World Health Organization Disability Assessment Schedule

**Column percentages may not add to 100% due to missing responses

Of the pre-SCI characteristics for the 6-month interview participants, the majority were male (76%), European (67%), married or living with a partner (54%), employed for ≥ 30 h per week (78%), and satisfied with their life (90%). The majority of participants also had no problems with their HRQoL (EQ-5D-3L) [33].

For the SCI-related characteristics, most participants (53%) had injuries of the least severe grade (AIS D), followed by 31% with the most severe grade (AIS A). Most SCIs were traumatic (77%); 50% of participants always used a wheelchair, while 12% only sometimes and 38% never used a wheelchair. Support from ACC was received by 79% of participants.

For the 6-month post-SCI characteristics, there was considerable worsening in the EQ-5D-3L HRQoL dimensions compared to pre-SCI. Most participants reported problems with mobility (92%), self-care (66%), usual activities (90%), and pain or discomfort (86%), compared to 10%, 3%, 7%, and 19%, respectively, pre-SCI. A smaller proportion of participants had problems with anxiety or depression (49%) and cognitive function (43%) 6 months post-SCI, but still higher than pre-SCI (12% and 9%, respectively). Most participants were also experiencing considerable disability 6 months post-SCI (80%) and the average number of SHCs was six (mean = 6; SD = 3; range = 0–12).

### Life satisfaction at 18 months and 10 years post-SCI

Of the 103 participants at 18 months, 68 (67%) were satisfied with life and 34 (33%) were not satisfied. One participant did not respond to the life satisfaction question so was excluded from analyses. Of the 63 participants at 10 years, 49 (78%) were satisfied with life and 14 (22%) were not. Thirty-six participants (57%) were satisfied at both timepoints (18 months and 10 years post-SCI). Overall, a greater proportion of participants were satisfied at 10 years (78%) compared to 18 months (67%). However, the percentage of participants not satisfied with life 10 years post-SCI (22%) was still higher than pre-SCI (10%).

### Univariable results at 18 months and 10 years post-SCI

Results from the univariable analyses assessing associations between potential predictors and life satisfaction outcomes at 18 months and 10 years are presented in Table 2.

**Table 2:** Univariable analyses of associations between characteristics and life satisfaction outcomes at 18 months and 10 years post-SCI

Characteristics	Life Satisfaction 18 months post-SCI			Life Satisfaction 10 years post-SCI		
	Satisfied (*n* = 68) *n* (%*)	Not Satisfied (*n* = 34) *n* (%*)	*p* Value	Satisfied (n = 49) n (%*)	Not Satisfied (*n* = 14) *n* (%*)	*p* Value
**Pre-SCI Characteristics**						
Age at onset of SCI (years)						
16–24	16 (84.2)	3 (15.8)	0.378	7 (70.0)	3 (30.0)	0.207
25–34	11 (64.7)	6 (35.3)		8 (61.5)	5 (38.5)	
35–44	12 (60.0)	8 (40.0)		6 (66.7)	3 (33.3)	
45–54	13 (56.5)	10 (43.5)		15 (88.2)	2 (11.8)	
55–64	16 (69.6)	7 (30.4)		13 (92.9)	1 (7.1)	
Sex						
Male	50 (64.1)	28 (35.9)	0.322	36 (76.6)	11 (23.4)	0.699
Female	18 (75.0)	6 (25.0)		13 (81.2)	3 (18.8)	
Ethnicity						
Māori	13 (81.2)	3 (18.8)	0.467	5 (71.4)	2 (28.6)	0.046
Pacific Peoples	4 (57.1)	3 (42.9)		0 (0.0)	2 (100.0)	
Asian	4 (80.0)	1 (20.0)		2 (66.7)	1 (33.3)	
European	47 (63.5)	27 (36.5)		42 (82.4)	9 (17.6)	
Relationship status						
Married/Living with partner	40 (66.7)	20 (33.3)	0.498	31 (79.5)	8 (20.5)	0.888
Single	21 (72.4)	8 (27.6)		11 (73.3)	4 (26.7)	
Separated/Divorced/Widowed	7 (53.8)	6 (46.2)		7 (77.8)	2 (22.2)	
Living arrangements**						
With family	51 (65.4)	27 (34.6)	0.86	40 (78.4)	11 (21.6)	0.554
With non-family	10 (66.7)	5 (33.3)		6 (85.7)	1 (14.3)	
Alone	6 (75.0)	2 (25.0)		3 (60.0)	2 (40.0)	
Highest educational qualification**						
No qualification	21 (77.8)	6 (22.2)	0.279	11 (84.6)	2 (15.4)	0.744
School	14 (70.0)	6 (30.0)		11 (78.6)	3 (21.4)	
Post-secondary	32 (60.4)	21 (39.6)		26 (74.3)	9 (25.7)	
Paid employment						
≥ 30 Hours	51 (64.6)	28 (35.4)	0.265	39 (76.5)	12 (23.5)	0.722
< 30 Hours	5 (100.0)	0 (0.0)		2 (100.0)	0 (0.0)	
Not in paid employment	12 (66.7)	6 (33.3)		8 (80.0)	2 (20.0)	
Job security**						
Very secure/secure	49 (65.3)	26 (34.7)	0.368	36 (75.0)	12 (25.0)	0.254
Insecure/very insecure	5 (83.3)	1 (16.7)		4 (100.0)	0 (0.0)	
Adequacy of household income**						
More than enough/enough	51 (68.0)	24 (32.0)	1	41 (82.0)	9 (18.0)	0.114
Just enough/not enough	17 (68.0)	8 (32.0)		8 (61.5)	5 (38.5)	
Prior chronic conditions						
No	42 (71.2)	17 (28.8)	0.257	24 (68.6)	11 (31.4)	0.049
Yes	26 (60.5)	17 (39.5)		25 (89.3)	3 (10.7)	
Prior injuries**						
No	56 (68.3)	26 (31.7)	0.667	38 (76.0)	12 (24.0)	0.585
Yes	12 (63.2)	7 (36.8)		10 (83.3)	2 (16.7)	
Prior long-term disability**						
No	59 (68.6)	27 (31.4)	0.512	44 (78.6)	12 (21.4)	0.668
Yes	9 (60.0)	6 (40.0)		5 (71.4)	2 (28.6)	
Family/whānau involvement						
Very large part/large part	63 (68.5)	29 (31.5)	0.239	46 (79.3)	12 (20.7)	0.319
Small part/very small part	5 (50.0)	5 (50.0)		3 (60.0)	2 (40.0)	
Pre-SCI life satisfaction						
Satisfied	63 (67.7)	30 (32.3)	0.459	45 (77.6)	13 (22.4)	0.901
Not satisfied	5 (55.6)	4 (44.4)		4 (80.0)	1 (20.0)	
EQ-5D-3L Mobility						
No problems	63 (69.2)	28 (30.8)	0.114	46 (79.3)	12 (20.7)	0.319
Any problems	5 (45.5)	6 (54.5)		3 (60.0)	2 (40.0)	
EQ-5D-3L Self-Care						
No problems	66 (66.7)	33 (33.3)	1	49 (79.0)	13 (21.0)	0.059
Any problems	2 (66.7)	1 (33.3)		0 (0.0)	1 (100.0)	
EQ-5D-3L Usual Activities						
No problems	64 (67.4)	31 (32.6)	0.58	47 (79.7)	12 (20.3)	0.167
Any problems	4 (57.1)	3 (42.9)		2 (50.0)	2 (50.0)	
EQ-5D-3L Pain or Discomfort						
No problems	55 (67.9)	26 (32.1)	0.603	42 (82.4)	9 (17.6)	0.072
Any problems	13 (61.9)	8 (38.1)		7 (58.3)	5 (41.7)	
EQ-5D-3L Anxiety or Depression						
No problems	61 (67.0)	30 (33.0)	0.821	44 (77.2)	13 (22.8)	0.731
Any problems	7 (63.6)	4 (36.4)		5 (83.3)	1 (16.7)	
EQ-5D-3L 0–100 VAS						
Mean ± SD	86.2 ± 18.0	84.0 ± 18.0	0.564	86.6 ± 12.8	81.4 ± 24.1	0.287
Cognitive function						
No problems	63 (67.0)	31 (33.0)	0.795	46 (79.3)	12 (20.7)	0.319
Any problems	5 (62.5)	3 (37.5)		3 (60.0)	2 (40.0)	
**SCI-related Characteristics**						
AIS grade						
A	14 (43.7)	18 (56.3)	0.004	11 (68.8)	5 (31.2)	0.226
B	4 (66.7)	2 (33.3)		1 (50.0)	1 (50.0)	
C	6 (60.0)	4 (40.0)		5 (62.5)	3 (37.5)	
D	44 (81.5)	10 (18.5)		32 (86.5)	5 (13.5)	
SCI aetiology						
Injury	54 (68.4)	25 (31.6)	0.243	40 (83.3)	8 (16.7)	0.101
Illness	11 (55.0)	9 (45.0)		8 (57.1)	6 (42.9)	
Don’t know	3 (100.0)	0 (0.0)		1 (100.0)	0 (0.0)	
Wheelchair usage						
Yes	26 (51.0)	25 (49.02)	0.004	17 (65.4)	9 (34.6)	0.14
No	32 (82.1)	7 (17.9)		26 (86.7)	4 (13.3)	
Sometimes	10 (83.3)	2 (16.7)		6 (85.7)	1 (14.3)	
ACC support						
Yes	56 (69.1)	25 (30.9)	0.299	42 (84.0)	8 (16.0)	0.02
No	12 (57.1)	9 (42.9)		7 (53.8)	6 (46.2)	
**6-month post-SCI Characteristics**						
Job security**						
Very secure/secure	28 (77.8)	8 (22.2)	0.072	23 (82.1)	5 (17.9)	0.573
Insecure/very insecure	11 (73.3)	4 (26.7)		7 (70.0)	3 (30.0)	
Not applicable	15 (51.7)	14 (48.3)		9 (69.2)	4 (30.8)	
EQ-5D-3L Mobility						
No problems	7 (87.5)	1 (12.5)	0.193	7 (87.5)	1 (12.5)	0.479
Any problems	61 (64.9)	33 (35.1)		42 (76.4)	13 (23.6)	
EQ-5D-3L Self-Care						
No problems	33 (89.2)	4 (10.8)	< 0.001	22 (84.6)	4 (15.4)	0.274
Any problems	35 (53.8)	30 (46.2)		27 (73.0)	10 (27.0)	
EQ-5D-3L Usual Activities						
No problems	11 (100.0)	0 (0.0)	0.013	5 (83.3)	1 (16.7)	0.731
Any problems	57 (62.6)	34 (37.4)		44 (77.2)	13 (22.8)	
EQ-5D-3L Pain or Discomfort						
No problems	9 (69.2)	4 (30.8)	0.834	7 (87.5)	1 (12.5)	0.479
Any problems	59 (66.3)	30 (33.7)		42 (76.4)	13 (23.6)	
EQ-5D-3L Anxiety or Depression**						
No problems	45 (83.3)	9 (16.7)	< 0.001	28 (82.4)	6 (17.6)	0.306
Any problems	23 (48.9)	24 (51.1)		20 (71.4)	8 (28.6)	
EQ-5D-3L 0–100 VAS						
Mean ± SD	60.3 ± 20.4	46.1 ± 19.8	0.001	56.8 ± 21.5	53.6 ± 19.0	0.611
Cognitive function						
No problems	45 (72.6)	17 (27.4)	0.115	28 (73.7)	10 (26.3)	0.335
Any problems	23 (57.5)	17 (42.5)		21 (84.0)	4 (16.0)	
Disability (WHODAS)						
No/lesser disability (0–9)	22 (100.0)	0 (0.0)	< 0.001	14 (93.3)	1 (6.7)	0.097
Considerable disability (≥10)	46 (57.5)	34 (42.5)		35 (72.9)	13 (27.1)	
SCI-related SHCs						
Mean ± SD	5.0 ± 2.8	7.4 ± 2.3	< 0.001	5.9 ± 2.6	6.9 ± 2.9	0.214
Satisfaction with social support						
Mean ± SD	22.4 ± 2.9	20.1 ± 4.3	0.002	21.9 ± 3.5	20.2 ± 4.7	0.153
Sexual activity decreased**						
No	21 (95.5)	1 (4.5)	0.005	11 (84.6)	2 (15.4)	0.566
Yes	44 (57.9)	32 (42.1)		36 (75.0)	12 (25.0)	
Not applicable	2 (66.7)	1 (33.3)		2 (100.0)	0 (0.0)	

*ACC* Accident Compensation Corporation; *AIS* American Spinal Injury Association Impairment Scale; *SCI* spinal cord injury; *SHCs* secondary health conditions; *VAS* Visual Analog Scale; *WHODAS* World Health Organization Disability Assessment Schedule

*Row percentages are presented

**Column percentages may not add to 100% due to missing responses

### Univariable associations with life satisfaction at 18 months post-SCI

There were no statistically significant differences (*p* > 0.05) for pre-SCI characteristics between those satisfied and not satisfied at 18 months post-SCI (Table 2).

Of the SCI-related characteristics, AIS grade and wheelchair usage were associated with life satisfaction (*p* < 0.05). The association with AIS grade indicates that greater proportion of those with a lesser SCI severity (AIS D) were satisfied with life at 18 months post-SCI compared to those with a greater SCI severity (AIS A). A greater proportion of people who never or only sometimes used a wheelchair were satisfied at 18 months post-SCI compared to those who always used a wheelchair.

Many 6-month post-SCI characteristics were associated with life satisfaction at 18 months post-SCI (*p* < 0.05). Higher proportions of participants were satisfied at 18 months if they had no problems with EQ-5D-3L self-care, usual activities, or anxiety or depression, had a higher self-rating of EQ-5D-3L VAS health, were experiencing no or lesser disability, had fewer SHCs, and had greater satisfaction with social support, and sexual activity was not reduced.

### Univariable associations with life satisfaction at 10 years post-SCI

Of the pre-SCI characteristics, at 10 years post-SCI significant associations with life satisfaction were found for ethnicity and prior chronic conditions (Table 2). Greater proportions of those of European ethnicity were satisfied compared to Māori, Pacific or Asian participants (*p* = 0.046). Also, greater proportions of participants were satisfied at 10 years post-SCI if they experienced a pre-SCI chronic condition compared to those without a prior chronic condition (*p* = 0.049). However, the very small number of participants in some cells in Table 2 may have influenced the true significance of these 10-year findings.

For SCI-related characteristics, only ACC support was associated with life satisfaction at 10 years post-SCI. Greater proportions of people receiving ACC support were satisfied compared to those not receiving ACC support (*p* = 0.020).

No statistically significant associations were found between 6-month post-SCI characteristics and life satisfaction 10 years post-SCI.

### Variables associated with satisfaction at 18 months and 10 years post-SCI as identified by multivariable modelling

Multivariable mini-models for assessing associations between four groups of variables and life satisfaction at 18 months and 10 years post-SCI are shown in Table 3. *p *value-based variable selection was not undertaken for the characteristics in Mini-Model 1 (age, sex, ethnicity, and pre-SCI life satisfaction) due to their importance and relevance to the outcome of interest.

**Table 3:** Multivariable mini-models of characteristics associated with being satisfied with life at 18 months and 10 years post-SCI

	18 Months post-SCI			10 Years post-SCI		
	Relative risk (satisfaction)	95% CI	*p *Value	Relative risk (satisfaction)	95% CI	*p *Value
**Mini-Model 1: demographic and pre-SCI characteristics**						
Age at onset of SCI (years)						
16–34	Ref			Ref		
35–64	0.82	0.63–1.07	0.149	1.32	0.95–1.84	0.095
Sex						
Male	Ref			Ref		
Female	1.19	0.90–1.59	0.225	1.08	0.81–1.43	0.596
Ethnicity						
Non-Māori	Ref			Ref		
Māori	1.31	0.99–1.73	0.063	0.92	0.60–1.42	0.701
Pre-SCI life satisfaction						
Satisfied	Ref			Ref		
Not satisfied	0.81	0.47–1.39	0.438	1.14	0.78–1.69	0.497
**Mini-Model 2: SCI-related Characteristics**						
Wheelchair usage						
Yes	Ref			Ref		
No/sometimes	1.62	1.20–2.18	0.002	1.3	0.96–1.76	0.087
ACC support						
Yes	–	–	–	Ref		
No				0.65	0.39–1.10	0.107
**Mini-Model 3: 6-month post-SCI EQ-5D-3L Characteristics**						
EQ-5D-3L Self-Care post-SCI						
No problems	Ref			–	–	–
Any problems	0.72	0.56–0.92	0.009			
EQ-5D-3L anxiety or depression post-SCI						
No problems	Ref			–	–	–
Any problems	0.68	0.50–0.93	0.017			
EQ-5D-3L 0–100 VAS post-SCI*	1.01	1.00–1.01	0.11	–	–	–
**Mini-Model 4: other 6-month post-SCI characteristics**						
Disability post-SCI (WHODAS)						
No/lesser disability (0–9)	Ref			Ref		
Considerable disability (≥10)	0.74	0.60–0.91	0.004	0.78	0.63–0.97	0.028
SCI-related SHCs*	0.94	0.90–0.98	0.006	–	–	–
Satisfaction with social support post-SCI*	1.05	1.00–1.11	0.057	–	–	–

Variable selection was not done in Mini-Model 1 for both 18-month and 10-year post-SCI timepoints due to their importance and relevance to the outcome of interest, so are retained regardless of their p value. For Mini-Model 2, AIS grade, SCI aetiology, and ACC support were considered for the 18-month model but were not retained, and AIS grade and SCI aetiology were considered at 10 years but not retained in the model. For Mini-Model 3, EQ-5D-3L mobility post-SCI, EQ-5D-3L usual activities post-SCI, and EQ-5D-3L pain post-SCI were considered but not retained in the 18-month model, and none of the 6-month post-SCI EQ-5D-3L characteristics were retained in the model at 10 years post-SCI. For Mini-Model 4, job security post-SCI, cognitive function after SCI, and sexual activity decreased were considered but not retained in the model at 18 months post-SCI, with SCI-related SHCs and satisfaction with social support post-SCI also not being retained at 10-year model

ACC, Accident Compensation Corporation; SCI, spinal cord injury; SHCs, secondary health conditions; VAS, Visual Analog Scale; WHODAS, World Health Organization Disability Assessment Schedule

*Continuous variable so the relative risk is for a one unit increase of the variable

–Variable not retained in the mini-model due to a *p *value ≥ 0.15

### Multivariable mini-model associations with life satisfaction at 18 months post-SCI

Māori participants were 1.31 times as likely to be satisfied at 18 months post-SCI than non-Māori. However, the 95% confidence interval (CI) indicates that this finding could be due to chance (95% CI: 0.99–1.73).

After considering other SCI-related variables, those who never or only sometimes used a wheelchair were 1.62 (95% CI: 1.20–2.18) times as likely to be satisfied at 18 months post-SCI than those who always used a wheelchair.

According to 6-month post-SCI characteristics, participants who reported any problems with EQ-5D-3L self-care were less likely (RR = 0.72; 95% CI: 0.56–0.92) to be satisfied at 18 months post-SCI than those with no problems. Similarly, participants who reported any problems with anxiety or depression were less likely (RR = 0.68; 95% CI: 0.50–0.93) to be satisfied at 18 months post-SCI than those with no problems.

Participants experiencing greater disability were less likely (RR = 0.74; 95% CI: 0.60–0.91) to be satisfied with life at 18 months post-SCI compared to those with no/lesser disability. Each additional SHC reported was associated with a 6% reduction in satisfaction with life at 18 months post-SCI (RR = 0.94; 95% CI: 0.90–0.98).

### Multivariable mini-model associations with life satisfaction at 10 years post-SCI

Participants who were older at SCI onset (35–64 years) were more likely (RR = 1.32; 95% CI: 0.95–1.84) to be satisfied 10 years post-SCI than younger participants (16–34 years). However, chance could also be an explanation for this finding.

Considering SCI-related characteristics, the association with wheelchair usage suggests that there is an increased likelihood of satisfaction at 10 years post-SCI for participants who never or only sometimes used a wheelchair (RR = 1.30; 95% CI: 0.96–1.76) compared to those who always used a wheelchair.

None of the 6-month EQ-5D-3L characteristics were retained due to all variables producing p values above the threshold (*p* > 0.15).

Participants who reported experiencing considerable disability were less likely (RR = 0.78; 95% CI: 0.63–0.97) to be satisfied at 10 years post-SCI compared to participants experiencing no/lesser disability.

### Multivariable final-model associations with life satisfaction at 18 months post-SCI

Table 4 presents the final multivariable model for life satisfaction at 18 months post-SCI. Sex was considered but then dropped from the final model due to its high *p *value in the mini-models and to avoid the possibility of over-fitting the data due to the small sample size. A 10-year model was unable to be developed due to the small sample size.

**Table 4:** Multivariable analysis of pre-SCI and post-SCI characteristics associated with being satisfied with life at 18 months post-SCI

	Relative Risk (Satisfaction)	95% CI	*p *Value
Age at onset of SCI (years)			
16–34	Ref		
35–64	0.89	0.70–1.12	0.310
Ethnicity			
Non-Māori	Ref		
Māori	1.17	0.91–1.50	0.224
Pre-SCI life satisfaction			
Satisfied	Ref		
Not satisfied	0.95	0.58–1.56	0.847
Wheelchair usage			
Yes	Ref		
No/sometimes	1.25	0.91–1.71	0.172
EQ-5D-3L Anxiety or Depression post-SCI			
No problems	Ref		
Any problems	0.75	0.54–1.03	0.074
Disability post-SCI (WHODAS)			
No/lesser disability (0–9)	Ref		
Considerable disability (≥ 10)	0.86	0.68–1.10	0.223
SCI-related SHCs*	0.95	0.90–0.99	0.021
Satisfaction with social support post-SCI*	1.03	0.98–1.09	0.252

Variables forced into this model include age, ethnicity, and pre-SCI life satisfaction

*SCI* spinal cord injury; *SHC*s secondary health conditions; *WHODAS* World Health Organization Disability Assessment Schedule

*Continuous variable so the relative risk is for a one unit increase of the variable

While adjusting for age, ethnicity, pre-SCI life satisfaction, and other explanatory variables, only SCI-related SHCs showed an association with 18-month life satisfaction. This indicated that participants experienced a 5% reduction in satisfaction at 18 months post-SCI with each additional SHC that they experienced (RR = 0.95; 95% CI: 0.90–0.99).

There is also a suggestion that participants with any problems with anxiety or depression at 6 months post-SCI were less likely to be satisfied at 18 months post-SCI than participants with no problems (RR = 0.75; 95% CI: 0.54–1.03); however, this finding could be due to chance.

## Discussion

Life satisfaction improved with time since SCI; a higher percentage of participants were satisfied with life 10 years post-SCI (78%) compared to 18 months (67%). This aligns with a Canadian 45-year longitudinal study that reported the years spent non-satisfactorily was a relatively small proportion of time compared to the years spent satisfactorily [41].

At 18 months post-SCI, participants who never or sometimes used a wheelchair, had no problems with self-care or anxiety or depression, experienced no or lesser disability, or had fewer SHCs were more likely to be satisfied with life. Strongest associations with life satisfaction at 18 months were found for anxiety or depression and SHCs. At 10 years post-SCI, only participants who experienced no or lesser disability were more likely to be satisfied with life. The univariable association between ACC support and life satisfaction at 10 years was not found in the mini-multivariable model at 10 years. It seems likely our study was underpowered to detect a relationship. Previous research with this cohort identified differences in socioeconomic outcome according to provision of ACC support [42]. Further research investigating ACC support and longer-term life satisfaction seems warranted.

Wheelchair usage does not appear to have been explicitly analysed in relation to post-SCI life satisfaction previously. The association found here is worth considering in the future research, particularly as the disabling effects of having to use a wheelchair are likely to exacerbate the issues of isolation and exclusion [43].

While no previous published research appears to have specifically examined the relationship between self-care and life satisfaction post-SCI, studies have investigated the related concept of functional independence. Cross-sectional and longitudinal studies from the Netherlands and the United States have found an increase in life satisfaction with increasing functional status and ability to do things independently [19, 44, 45]. These challenges with self-care and functional independence were also identified in a NZ study after the transition from spinal unit to the ‘real world’ [46]. These participants felt like the rehabilitation centre did not appropriately prepare them for their return to the community post-SCI and hence struggled with their reintegration and ability to cope [46]. This aligns with our finding of the higher likelihood of being satisfied when experiencing no problems with self-care.

Findings, similar to ours, between anxiety or depression and life satisfaction were identified in a Swedish study where problems with anxiety or depression were associated with lower life satisfaction [47]. Similarly, three other cross-sectional studies from Canada, the Netherlands and the United Kingdom found having fewer problems with psychological functioning was associated with higher life satisfaction [13, 48, 49]. Future research using specific measures of mental health outcomes seems warranted.

Disability was found to be significantly associated with life satisfaction at both 18 months and 10 years post-SCI in the mini-model analyses. This indicates its long-term importance among this cohort, with other studies showing similar associations between disability and life satisfaction post-SCI [44, 45]. These include a cross-sectional study of 190 people in the United States [44], and a five-year prospective cohort study from the Netherlands [45], which found that increased physical disability was associated with decreased life satisfaction.

Our study provides further evidence to support the association between the presence of SHCs and lower life satisfaction, as identified in studies from the Netherlands and Sweden [50–52]. Additionally, our study also found that the greater the number of SHCs experienced, the lower the level of life satisfaction [50–52]. Previous research with this cohort has explored SHCs post-SCI [26]; however, further in-depth research is needed to investigate SHCs in relation to life satisfaction—particularly as many SHCs are preventable.

Our study contributes important findings about life satisfaction outcomes 10 years post-SCI and the areas where interventions and support are needed to improve life satisfaction. This is particularly important as it is the first study to examine life satisfaction among those with a SCI in NZ. While the majority of participants expressed being satisfied with life post-SCI, it is still important to address and support those who are at risk of lower life satisfaction.

The large number of variables included in the study means that life satisfaction was able to be assessed against a wide range of aspects from all areas of life. The inclusion of a 10-year follow-up period is a strength of this research as there have been few studies on this topic longitudinally following up the same participants from the onset of SCI to 10 years post-SCI and beyond [17, 20, 41, 53–55]. The considerable work and input from people with lived experience of SCIs into the design and development of the study and interview questions to ensure they were applicable and relevant, as well as interviewers with lived experience of SCI, are further important strengths of this study.

This research may be prone to recall bias due to the pre-SCI characteristics being collected in an interview 6 months post-SCI. This could have caused participants to over- or under-estimate pre-SCI characteristics depending on their post-SCI status. However, the SCI is likely to have provided a clear ‘anchor point’ from which to recall prior characteristics, reducing recall bias, and previous research with injured New Zealanders suggests recall bias is likely to be minimal [56].

While this study was the largest undertaken in NZ and included 118 participants at baseline, the sample size was small, particularly at 10 years post-SCI. The number of participants at the 10-year timepoint was reduced due to participants changing contact details and being uncontactable, as opposed to declining participation (only one person declined). This made 10-year analyses difficult as only a limited number of variables could be included in the multivariable analyses. However, this analysis was adapted to suit the small sample size and the available resources. Future research including a larger sample size would be valuable to investigate these findings further. Further research is also needed on older adults and young people with SCIs.

Of the 118 participants recruited to the study, 15 (13%) were lost to follow-up at 18 months, and 63 participants (53%) were followed up 10 years post-SCI. A greater proportion of Māori were lost to follow-up at 18 months (*p* = 0.004) and 10 years (*p* = 0.014). Also at 10 years, a higher proportion of participants who always used a wheelchair were lost to follow-up (*p* = 0.042). Further research should be conducted to assess the relationships between life satisfaction and SCI for Māori, and life satisfaction and wheelchair usage.

Disability at 6 months post-SCI was associated with life satisfaction at both 18 months and 10 years post-SCI. Efforts from healthcare providers and rehabilitation centres should focus on work with individuals to minimise the experiences or impacts of disability. This could be done by organising appropriate mobility aids, home help, or vocational rehabilitation. Additionally, policy makers should implement regulations around accessible environments, improved public transport, and community participation, while further work should be done on reducing disability stigma and stereotypes to create a more accessible and supportive environment.

## Conclusion

This research has found that most people were satisfied with their lives after a SCI, with a higher proportion of participants being satisfied 10 years  on compared to 18 months post-SCI. However, more work is needed to ensure that those who were not satisfied with life are able to receive the support they need to improve their level of satisfaction.

This study has identified experiences of disability and SHCs 6 months post-SCI as key areas to improve to increase the proportion of people experiencing satisfaction with life, in both the short and longer terms.

## Data Availability

Data use is restricted due to the Study Participant Information Sheet and Consent Form, which explicitly state collected data will not be shared publicly. Qualified researchers, and postgraduate students, may request to collaborate with the research team by contacting the corresponding author.
